# First‐In‐Human, Single Ascending Dose and Food‐Effect Study in Healthy Volunteers of SUL‐238, a Novel Orally Administered Mitochondria‐Directed Drug Candidate

**DOI:** 10.1002/alz70859_100985

**Published:** 2025-12-25

**Authors:** Nadir Ulu, Daniël Henri Swart, Zafer Sezer, Mustafa Mümtaz Mazıcıoğlu, Ahmet İnal, Sedat Altuğ, Serhat Kozlu, Adrianus Cornelis van der Graaf, Robert Henk Henning, Guido Krenning, Murat Emre

**Affiliations:** ^1^ Gen İlaç ve Sağlık Ürünleri A.Ş., Ankara Turkey; ^2^ University Medical Center Groningen, Groningen Netherlands; ^3^ Sulfateq B.V., Groningen Netherlands; ^4^ Erciyes University, Kayseri Turkey; ^5^ Hakan Cetinsaya Good Clinical Practice and Research Centre (İKUM), Kayseri Turkey; ^6^ Demiroğlu Bilim University, İstanbul Turkey; ^7^ Istanbul University, Istanbul Turkey

## Abstract

**Background:**

There is compelling evidence that mitochondrial dysfunction may be crucial in the pathophysiology of Alzheimer’s disease (AD) and Parkinson’s disease (PD). SUL‐238 is a novel, investigational small molecule which improves mitochondrial function in animal models. Its oral administration (partially) restores AD‐associated changes in protein expression in an AD mouse model and results in a significant reduction in amyloid plaque size/accumulation, enhancement of synaptic transmission and improved memory performance. These findings indicate that modulating mitochondrial metabolism by SUL‐238 may provide a promising approach for treatment of AD.

**Method:**

This was a single, oral ascending dose (SAD) Phase 1, first‐in‐human, randomized, double‐blind, placebo‐controlled study conducted in healthy adults (ClinicalTrials.gov identifier, NCT06277492). Part 1 included 6 cohorts (50, 100, 250, 500, 1000 and 2000 mg orally, n=23). In part 2, pharmacokinetics (PK) of a single 1000 mg oral dose was investigated in 10 healthy adults. In Part 2B, food effect was assessed using a randomized, single oral 2000 mg dose, two‐treatment, two‐period, crossover design (n=20). The primary objectives were assessment of safety, tolerability, and PK. Cerebrospinal fluid (CSF)‐to‐plasma concentration percentage was determined in the SAD and food effect parts.

**Result:**

There were no adverse effects (AEs) that precluded dose escalation, AE rates were comparable between participants receiving SUL‐238 and placebo. All AEs were mild or moderate in severity. Mean t_1/2_ ranged between 0.86‐3.80 hours and mean T_max_ ranged between 0.50‐1.39 hours. Under fed condition there was a 50% reduction in C_max_ and a 60% reduction in AUC_0‐inf_ as compared to fasting condition. The mean CSF‐to‐plasma percentages at 2‐ and 8‐hours post‐dose were 21.1% and 74.2%, respectively.

**Conclusion:**

In this Phase 1, first‐in‐human, healthy volunteer study, 50‐2000 mg single oral doses of SUL‐238 were safe and well‐tolerated, while demonstrating a favourable PK profile, and a high CSF penetration.

